# In vitro and in vivo investigation of a thyroid hormone system-specific interaction with triazoles

**DOI:** 10.1038/s41598-024-55019-3

**Published:** 2024-03-18

**Authors:** Asya Kadic, Patricia Oles, Benjamin Christian Fischer, Anne Elisabeth Reetz, Boubacar Sidiki Sylla, Katreece Feiertag, Vera Ritz, Tanja Heise, Philip Marx-Stoelting, Tewes Tralau, Kostja Renko, Marize de Lourdes Marzo Solano

**Affiliations:** 1 Department of Pesticides Safety, German Federal Institute for Risk Assessment (BfR), Max-Dohrn-Str. 8-10, 10589 Berlin, Germany; 2 German Federal Institute for Risk Assessment (BfR), German Centre for the Protection of Laboratory Animals (Bf3R), Diedersdorfer Weg 1, 12277 Berlin, Germany; 3 Institute of Veterinary Pathology (WE12), Freie University Berlin, Berlin, Germany

**Keywords:** Thyroid hormone pathway, Endocrine disruption, Adverse outcome, Thyroid network, Pesticides, Systems biology, Endocrinology, Pathogenesis

## Abstract

Alterations in thyroid hormones (TH) and thyroid-stimulating hormone levels are frequently found following exposure to chemicals of concern. Dysregulation of TH levels can severely perturb physiological growth, metabolism, differentiation, homeostasis in the adult and developmental processes in utero. A frequently identified mode of action for this interaction is the induction of hepatic detoxification mechanisms (e.g. SULTs and UGTs), which lead to TH conjugation and elimination and therefore interfere with hormonal homeostasis, fulfilling the endocrine disruptors (EDs) definition. A short-term study in rats with dietary exposure to cyproconazole, epoxiconazole and prochloraz was conducted and hepatocyte hypertrophy, hepatic UGT activity and Phase 1/2 gene expression inductions were observed together with changes in TH levels and thyroid follicular hypertrophy and hyperplasia. To test for specific interaction with the thyroid hormone system, in vitro assays were conducted covering thyroidal I-uptake (NIS), TH transmembranal transport via MCT8 and thyroid peroxidase (TPO) function. Assays for iodothyronine deiodinases (DIO1–DIO3) and iodotyrosine deiodinase (DEHAL1) were included, and from the animal experiment, Dio1 and Dehal1 activities were measured in kidney and liver as relevant local indicators and endpoints. The fungicides did not affect any TH-specific KEs, in vitro and in vivo, thereby suggesting hepatic conjugation as the dominant MoA.

## Introduction

Thyroid hormones (TH) are crucial for fetal development, especially in the organization and maturation of neuronal structures, and they play an important role in homeostasis, rate of metabolism of lipids, carbohydrates and proteins, cardiovascular, neurological, and skeletal systems, immunity, and communication between other hormones^1–5^. Circulating TH levels are actively regulated and finetuned by TSH-secretion from the pituitary gland involved in a negative feedback loop and with a direct impact on thyroidal TH biosynthesis, but also depend on TH clearance (e.g. hepatic catabolism). Any interaction with central regulation, TH biosynthesis, metabolism (including activation and inactivation), humoral transport and cellular uptake may have a significant impact on the levels of TH available at the receptors of the target cells. Both the thyroid gland and the Hypothalamus-Pituitary-Thyroid (HPT) axis are primary targets of chemical substances in rodents, making them a focal point in toxicological studies investigating effects of chemicals.

Cyproconazole (2RS,3RS;2RS,3SR)-2-(4-chlorophenyl)-3-cyclopropyl-1-(1H-1,2,4-triazol-1-yl)butan-2-ol) is a triazole fungicide. The main target organ of cyproconazole is the liver after short-term to long-term exposure and it is recognized in the EU as toxic to reproduction with an observed increase in maternal liver weight and a slight increase in peri/pre- and postnatal mortality^6^. In rat and mouse liver enzyme studies, it strongly induces phase I and II enzymes in rats, including NCPR, CYP1A, GST, and UDPGT in mice^7^. It is also directly involved in hepatic, toxin-induced receptor induction, including nuclear receptor constitutive androstane receptor (CAR), which was induced in a hCAR/hPXR mice model after receiving cyproconazole^8,9^. This fungicide was originally approved for use as an active substance in plant protection products (PPP) and biocidal products (BP) in the EU in 2011, but its approval expired on May 31st, 2021^10^ and 31st October 2020 (Reg. (EU) 436/2014), respectively.

Epoxiconazole (2RS, 3SR)-1-[3-(2-chlorophenyl)-2,3-epoxy-2-(4-fluorophenyl)propyl]-1H-1,2,4-triazole is also a triazole fungicide from the azole family. Since its launch in 1993, it has been used by the BASF group as a pesticide, individually or in combination with other crop protection products, to control pests of cereals in more than 50 countries worldwide. Epoxiconazole may damage the unborn child and is suspected of damaging fertility (Repr. 1B), is toxic to aquatic life with long-lasting effects (Aquatic Chronic 2), and is suspected of causing cancer (Carc. 2)^11^*.* It was originally approved for use as an active substance in plant protection products (PPP) in the EU in 2009, but the authorization expired on April 30th, 2020. The use and sale of the pesticide was banned in France in 2019 due to potential harmful effects on human health^12^.

Prochloraz (*N*-propyl-*N*-[2-(2,4,6 trichlorophenoxy)ethyl]imidazole-1-carboxamide), is an imidazole fungicide that also belongs to the azole family. Adverse effects in the liver (critical increase in liver size and weight) were observed across all species in short-term studies with mice, rats, and dogs. Supplementary long-term studies showed that prochloraz could be considered as a phenobarbitone-type inducer of the hepatic mixed-function system of male rats and mice^13^. It was originally approved for use as an active substance in plant protection products (PPP) in the EU in 2012, but expired on December 31st, 2021^14^.

According to the Guidance from European Food Safety Authority (EFSA) and European Chemicals Agency (ECHA) for the identification of endocrine disruptors (ED) in biocides and plant protection products, there are three criteria that a chemical needs to fill to be considered as having ED properties: (1) It shows an adverse effect in an intact organism or its progeny or non-target organisms, which is a change in the morphology, physiology, growth, development, reproduction, or life span of an organism, system, or (sub)population that results in an impairment of functional capacity, an impairment of the capacity to compensate for additional stress or an increase in susceptibility to other influences; (2) it has an endocrine mode of action, i.e. it alters the function(s) of the endocrine system; (3) the adverse effect is a consequence of the endocrine mode of action (1 is a result of 2—a plausible link between the adverse effect and the endocrine activity)^15,16^. Thus, it is important to consider the adverse effects as well as the mode of action of a chemical of concern.

In 2019, EFSA has released a cumulative risk assessment (CRA) report in which they established cumulative assessment groups (CAGs) of active substances (AS) for two thyroid-related toxicity endpoints/effects; hypothyroidism and parafollicular cell (C-cell) hypertrophy, hyperplasia and neoplasia, induced by pesticides. 128 (out of 400) ASs were included in the CAG for hypothyroidism, and cyproconazole was one of them, amongst other azole fungicides^17^.

**Figure 1:**
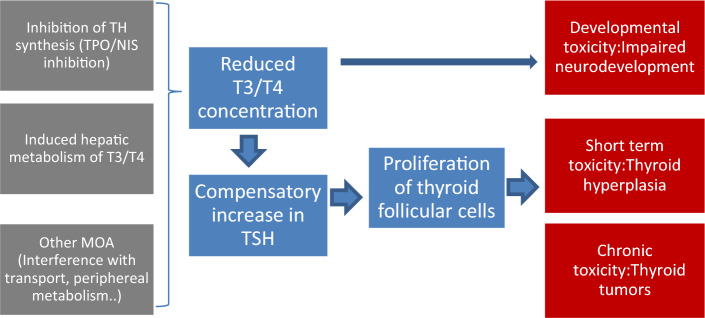
Human health adverse outcome pathway example in the thyroid.

There are several well established molecular initiating events (MIE) that may lead to adverse effects on the TH system^18^, such as inhibition of thyroid peroxidase (TPO), the sodium iodine symporter (NIS) or the deiodinases (DIO) (which would focus on TH synthesis and metabolism within thyroid or the peripheral TH), or induction of hepatic TH metabolism via nuclear receptors. Noyes et al. gives an overview on MIE, key events and adverse effects in line with the adverse outcome pathway concept^19^, that is summarized and condensed in Fig. 1.

In our current study, we have focused on the activation of xenobiotic receptors by azole fungicides which are known to upregulate the UGT (UDP-glucoronosyltransferase) enzymes. UGT enzyme increases the metabolism of circulating T3 and T4 by glucoronic acid conjugation and biliary excretion of the conjugated hormones. A decrease in T3 and T4 is therefore observed, which results in increase of TSH to compensate for the decreased TH levels. This increase in TSH levels can be attributed to the feedback on the HPT-axis and leads to thyroid histological changes (increased epithelial cell line, decreased colloid area, hyperplasia and hypertrophy).

A 28-day feeding study in male Wistar rats with cyproconazole (C), epoxiconazole (E) and prochloraz (P) at different dose groups was conducted^20,21^ and a significant increase in liver weight and hepatic enzyme induction was found in the treated groups. This confirmed the liver as a main target organ and the hepatotoxicity activation by interaction with the nuclear receptors CAR, pregnane X receptor (PXR) and aryl hydrocarbon receptor (AhR), all three included as MIE in the recently proposed AOP network for THS-related effects^19^.

In the present study, THS-specific interaction was observed in rats following a 28-day feeding exposure to cyproconazole, epoxiconazole and prochloraz with investigations of thyroid histology and morphometry, TH and TSH serum levels and TH metabolism induction via hepatic detoxification (e.g. Uridine 5ʹ-diphospho-glucuronosyltransferase-UGT activity assay). Additionally, a selection of MoA-focused in vitro assays dedicated to THS-specific interaction (including thyroidal I-uptake (NIS), thyroid peroxidase (TPO) and iodothyronine deiodinases (DIO1-3) inhibition) by cyproconazole, epoxiconazole and prochloraz were conducted. Furthermore, from the animal experiment, activities of Dio1 and Dehal1 were measured in kidney and liver in C-treated rats as potential local axis-related biomarkers, as suggested before^22^.

## Material and methods

### Test substances and chemicals

Hepatotoxic fungicide cyproconazole (C) (CAS no. 94361-06-5, Batch no. CHF1E00042, Purity 96.8%) was obtained from Syngenta (Basel, Switzerland). Epoxiconazole (E) (CAS no.133855-98-8, Batch no. 8563, Purity 97.0%) and prochloraz (P) (CAS no. 67747-09-5, Batch no. COD-000718, Purity 98.0%) were supplied by BASF (Ludwigshafen, Germany). Phenobarbital sodium (PB) (CAS no 57-30-7, Batch no 100823, Purity > 99%) was purchased from Sigma-Aldrich (Hamburg, Germany). Test substance was obtained directly from the manufacturers in technical grade (the same quality and purity of which the substance is used in plant protection products). Concentration and stability of test substance in the rodent diet, except for phenobarbital, was checked by SGS Fresenius (Berlin, Germany) by multi-method according to ASU L. 00.00-115 LC under GLP conditions^20^. T3 and T4 were obtained from Sigma-Aldrich. Chemicals and other material needed for the in vitro test systems were obtained from respective commercial sources and vendors (e.g. Sigma-Aldrich, Cayman Chemicals or Roth) in sufficient quality.

### Animal experiments

A 28-day feeding study with 9 weeks old male Wistar (Crl:Wi) rats exposed to the fungicides cyproconazole, epoxiconazole and prochloraz was performed as already described by Heise et al.^20^. The animal experiment was approved by the Committee for Animal Welfare of the City of Berlin at LaGeSo, approval # C 114-Reg 0262/11, on 20.11.12. LaGeSo is the competent authority in Berlin for all issues related to animal test approval in line with German Animal Protection Act (TierSchG). At the end of the treatment period, the animals were deeply anaesthetised with Sevofluran (Abbot, Germany), cardial blood was sampled by use of a 21G Sangocan blood sampling canule (Kabe, Germany) and animals were finally sacrificed in 95% CO_2_ and 5% O_2_. All experimental procedures were performed in accordance with the relevant ARRIVE guidelines and regulations. All methods were performed in accordance with the relevant guidelines and regulations.

In summary, 5 animals were assigned for each treatment group and 5 animals for the phenobarbital positive control group, as well as 6 untreated animals were used in total (see Table 2). Dose selection was based on ‘no observed adverse effect levels (NOAELs)’ in 28- and 90-day feeding studies which were conducted by the manufacturers up to a dose level showing clear effect dose (10 × NOAEL). For cyproconazole it was used 1000 ppm (ca. 74 mg/kg bw per day), for epoxiconazole 900 ppm (ca 62 mg/kg bw per day) and for prochloraz 1000 ppm (ca 68 mg/kg bw per day). Phenobarbital was used as a positive control and was administered in a dietary concentration of 500 ppm (ca 32 mg/kg bw per day), as it is known to cause hepatotoxicity (promotes liver tumors) without pronounced systemic effects^23^. Further experimental details and results are given elsewhere^20,24,25^.

The Organisation for Economic Co-operation and Development (OECD) TG407 guideline^26^ was followed as principles of the experiment and parameters, with the exception that the range of organs for histopathology was limited. In addition, only male animals were used, since males had been shown to be slightly more sensitive in previous studies on which approval of the respective fungicides was based^20^.

At necropsy, liver, kidney and thyroid (with trachea) were removed and stored frozen at − 80 °C until processing.

In this present publication, the scope is limited to triazoles effects in the thyroid tissue and liver microsomes activity, as other toxicity effects were already investigated somewhere else^9,20,21,24,25^. At these studies, cyproconazole, epoxiconazole and prochloraz were administered individually at five dose levels to male rats, ranging from slightly above the reference values to a clear toxic effect dose. In summary, there was no mortality, and all rats remained in good health throughout the study. Body weight and in particular weight gain were compromised by administration of all three test substances. At the lower dose levels, there were no adverse effects on body weight and its development, on food consumption or utilisation with any of the substances. There were no alterations on the clinical chemistry parameters. Liver weight and hepatocellular hypertrophy were identified mostly at the top dose of each pesticide.

### Microsomes isolation

Approximately 1 g of thawed rat liver were homogenized with 250 mM sucrose solution containing 1 mM EDTA using a Potter–Elvehjem tissue grinder (400 rpm) and kept cool during homogenization. The homogenate was subsequently centrifuged for 10 min at 2500 rpm (420×*g*) and 4 °C. In case a fatty layer formed it was removed carefully using a pipette. The microsome-containing supernatant was decanted into an ultracentrifuge tube and the pellet was discarded. The supernatant was centrifuged for 60 min at 38,000 rpm (97,000×*g*) and 4 °C in an ultra-centrifuge. The supernatant was discarded, and the pellet was washed by resuspending it with 150 mM KCl solution and again ultra-centrifuged for 60 min at 38,000 rpm (97,000×*g*) and 4 °C. Finally, the pellet was resuspended in 250 mM sucrose solution (without EDTA) and aliquots were taken for protein content determination or stored at − 80 °C for later use. Protein concentrations were determined with the bicinchoninic acid (BCA) assay (Sigma Aldrich, USA, P0914-5AMP, Lot SLBZ6885) with individual measurements (per animal) in a total of 5 animals per treatment group and using a Tecan Plate Reader and software (Tecan i-control, Infinite 200Pro).

### UGT activity assay

UGT activity analysis was conducted using UGT Activity Assay Ligand Screening Kit (BioVision, USA, K692-100, Lot 6F15K06920) and the protocol was followed according to the supplier. In summary, reaction mixes were added to 2 µg microsomes per treatment group per animal (n = 5) in duplicates and were incubated for 5 min at 37 °C protected from light. Finally, 5X UDPGA substrate was added to each sample well and fluorescence (Ex/Em = 415/502 nm) was immediately measured in kinetic mode for 40 min at 37 °C using a Tecan Plate Reader and software (Tecan i-control, Infinite 200Pro). One unit of UGT activity represents the amount of enzyme that glucuronidates 1 µmole of fluorescent substrate per minute (yielding a non-fluorescent conjugate) at 37 °C and pH 7.5.

### Serum TH and TSH measurements

Total TH levels (tT3, tT4) and TSH in rats after 28-days of treatment with cyproconazole, epoxiconazole, prochloraz, phenobarbital and non-treated animals (Control) were analysed by MagPix multiplex assays (MILLIPLEX^®^ Rat Thyroid Magnetic Bead Panel—Endocrine Multiplex, Cat # RTHYMAG-30K, Kit Lot # 3253455). Multiplex measurements from 25 µl serum per replicate (n = 2) by fluorescence were conducted using a Bio-Plex MAGPIX Multiplex Reader according to the manufacture instructions.

### Histopathology and microscopy

One thyroid lobe was bisected transversely at the level of the parathyroids, generating two sections anterior and posterior. Both sections were blocked using Tissue-Tek^®^ O.C.T. and frozen at − 80 °C again until ready for the standard histology analysis. Thyroid sections were trimmed from the block and slices with 7 µm thickness and prepared using a Cryostat HM550 from Thermo Scientific Cryotome. Three slides with two to three cuts of 7 µm from each thyroid section were prepared, with a total of six to nine thyroid tissue cuts per animal for microscopy analysis. The cuts were fixed with 10% Formalin and prepared using Haematoxylin and Eosin (H&E) staining standard protocol. H&E-stained thyroid slices were coded and evaluated under Zeiss Axio Observer D1 base with Camera Port Microscopy and halogen brightfield illumination type at magnifications from 50 to 200×. Pictures were captured by a digital camera and processed by Software Zeiss Zen—Digital Imaging for Light Microscopy. Sections were scored blindly by three observers and the histological nomenclature used was based on the Standardized System of Nomenclature and Diagnosis Criteria^27^, referenced to the corresponding negative (not treated) and positive (PB) control groups.

### Thyroid morphometry analysis

The stained sections were digitized using the Aperio CS2 Scanner (Leica biosystems Imaging Inc.) with a 400× magnification (equivalent to a resolution of 0.25 µm/pixel). The thyroid sections were digitally evaluated using the Aperio ImageScope × 64 software and the QuPath v0.4.3 software. For this, the areas to be analyzed were initially annotated. Initially for every treatment group 4–6 animals, several slides each containing 2–3 thyroid cuts were cut from the middle of the tissue. For every animal two cuts were evaluated. When possible, the morphometric analysis was focused on one of the central sections of the thyroid, representative of the whole lobe. Through annotating the tissue following values were derived: surface area of the whole thyroid, area of the high-power fields, non-thyroid tissue in high power fields, number of follicles within high power fields, area of follicles lumen within the high-power fields, epithelial height. All annotations were performed while blinded for treatment groups and by two observers, and only the most relevant are shown in the results (Fig. 5A–E). Below is the glossary and explanation, with mathematical derivation, of the key dimensions used (Table 1). Initially at Image Scope, in each scanned slide, the annotations were created based on four individual layers. Layer 1 outlined all thyroid tissue while at 15 × magnification. At layer 2, "axis + Grit" was selected and a magnification of 2 × was chosen to make annotations in all rectangles which consisted of more than 50% thyroid tissue. Randomly selected rectangles were marked to the size of the grid under double magnification using the "rectangle tool" and are called “high power fields”. Therefore, the analyzed tissue in the next step had approximately the same size. Subsequently, areas that were not thyroid tissue were also annotated using the "negative pen tool", such as intrathyroidal located parathyroid glands. After completion of editing, the size of the annotated areas and the length of the circumference was used for comparison between treatment groups. The sum of the positive rectangles and the negative non thyroid tissue was named L2 at the glossary (Table 1). In the next step, layer 3 was selected, and the color red was assigned. Subsequently, all follicular lumen at the border between the apical side of the epithelium and the lumen were annotated at a magnification of 20 × in order to determine the number of follicles and lumen surface area per high power field. The derived values were used for comparison between treatment groups. Subsequently, under layer 4, the "ruler tool" was used to measure the follicular epithelial height of individual cells with a 40 × magnification. At QuPath the selection of "Brightfield (H&E)" as image type was implemented and after preprocessing for visual staining quality, all thyroid tissue was marked using the 'Brush Tool' at a 20 × magnification. This marked tissue was then prepared for further processing using an automatic cell counter under the same settings for every slide.

**Table 1:** Morphometry glossary and annotations key.

Name	Description	Derivation
Epithelial cell area	The effective area of epithelial cells	L2 − L3
Nuclear count by area	Nuclei by thyroid area	Nuclear Count / L1 − QuPath
Number of follicles	Number of annotated follicle lumen in L2	# of L3 annotations
Epithelial cell area by num. of follicles	Cell area per follicle	(L2 − L3) / # of Follicles
Epithelial cell area by follicle lumen area	Ratio between the epithelial cell area and the follicle lumen area. Also referred to as ‘Thyroid activation index’	(L2 − L3) / L3

### Dio1 and Dehal1 from ex vivo tissue

Briefly, chunks of liver or kidney were powdered in the frozen state, using a bead mill (Mixer CryoMill Retsch) and continuous handling on dry ice and liquid nitrogen. The tissue powder was mixed with homogenization buffer (250 mM D-Sucrose, 20 mM Hepes, 1 mM EDTA, pH 7.4) and further disrupted by sonification (2 × 10 pulses, 100% amplitude, UP50H, Hielscher, Teltow, Germany). As described before^22,28^, homogenates were measured for protein concentration and uniform amounts of protein per reaction were used from Dio1 (40 µg) and Dehal1 (80 µg) measurements. To determine background activity for later subtraction, a pool of samples was used to setup four reactions per run containing 1 mM propy-6-thiouracil as well-known, specific Dio1 inhibitor, or dibromotyrosine, a Dehal1 inhibitor, respectively. Incubation time was two hours for Dio1 and four hours for Dehal1 determination. Subsequent steps for ex vivo samples were identical to the setup for compound testing, as described below or elsewhere^29^. For the conversion of photometric measurements from the Sandell-Kolthoff-reaction to absolute activities in “pmol (released iodide)/mg (protein) * min” an iodide standard curve was separately measured, from which the amount of released iodide was extrapolated, as described earlier^22,28^.

### In vitro assays

For the efficient use of the applied in vitro assays related to the thyroid hormone system, a dilution series of cyproconazole, epoxiconazole and prochloraz was prepared and applied. This involved the subsequent dilution from a master plate (10 × dilution series in DMSO, starting from 5 mm), with a final dilution step into the incubation media or reaction mixture, resulting in a DMSO concentration of 1% and maximal concentration of 50 µM of the respective test compounds in all assays. These range-finder experiments with broad concentration coverage (N = 2) were performed for the potential initial identification of strong and dose-dependent effects up to the maximum tested concentration of 50 µM.

### DIO1/2/3 activity

Testing for potential inhibition of DIO1, 2 and 3 activity was conducted as described here^30^. In brief, homogenates from cell lines with stable expression of the three isoenzymes (HEK293-hDIO1/2/3) were incubated together with the respective optimal substrates (10 µM, DIO1: rT3, DIO2: T4, DIO3: T3) under optimized buffer conditions with 40 mM DTT as cofactor at 37 °C for 2 h (DIO1, DIO3) or 4 h (DIO2), respectively under constant shaking (600 rpm) and in the presence of test compound, solvent control or positive control. Subsequently, the released iodide was separated and eluted using a cation exchanger (DOWEX 50wx2) and quantified via photometry, following the Iodine-catalyzed destaining of Ce in the Sandell-Kolthoff-reaction. Changes of absorption (415 nm) after 20 min were used to calculate individual activities in %. Well-known DIO-Inhibitors were used for DIO1 (propyl-6-thiouracil, 1 mM) and DIO2 (Xanthohumol 150 µM) and DIO3 (Xanthohumol, 500 µM) as positive control, defining 0% activity. 100% activity was defined by respective solvent controls (1% DMSO).

### DEHAL1

Azole-effects on DEHAL1 activity were tested as described here^31^. In brief, homogenates from a cell line (HEK293), transiently transfected with a respective expression plasmid, was incubated under optimized conditions, in the presence of monoiodotyrosine (10 µM) as substrate and at 37 °C for 2 h under constant shaking (600 rpm) in the presence of test compound, solvent control or positive control (dibromotyrosine (DBT), 1 mM). Subsequently, the released iodide was separated, eluted and quantified via photometry as described above. Changes of absorption (415 nm) after 20 min were used to calculate individual activities in %. Solvent control defined 100% activity level, while data from the DBT-treated samples were used to set 0% activity.

### NIS

A cell line (HEK293-hNIS-FLuc) with stable expression of a fusion protein of the human NIS and firefly-luciferase was used as a test system, to identify interaction of the test compound with the NIS-mediated iodide uptake into the cell. As described earlier^30^, cells were incubated for 30 min with hanks’ buffered salt solution containing 0.1% BSA and 10 µM sodiumiodide, in the presence of test compound, solvent control or positive control (sodiumperchlorate, 100 µM). Subsequently, cells were washed with PBS and cold ddH2O and relative amounts of intracellular iodide was determined via Sandell-Kolthoff-reaction, reflecting the uptake in %. Solvent control defined 100% uptake level, while data from the cells co-incubated with 100 µM perchlorate served to set the 0% uptake level.

### TPO

Cell extracts from FTC238-hrTPO cells^32^ with high expression of enzymatically active TPO were used to test for inhibitory effects of azoles on this key enzyme of TH biosynthesis, using AmplexUltraRed (ThermoFisher) as substrate as described by others^33,34^. In brief, a mastermix of enzyme, KPO4-buffer (100 mM, pH 7) and AmplexUltraRed (100 µM) was prepared and distributed on a black microtiter plate (80 µl/well). Test compounds, solvent control or positive control was added (10 µl), and the reaction was started by adding H2O2 (10 µl, 0.035%). After 15 min of incubation at 37 °C, fluorescence of the product was quantified at 535 nm/590 nm using a Genios fluorimetric platereader (TECAN). After subtraction of background signal, determined from the positive controls (methimazole (MMI), 1 mM), relative fluorescence units were directly converted into relative peroxidase activity in %. Data from the reaction mixtures co-incubated with 1 mM MMI served to set the 0% background signal, while the solvent control defined 100% TPO activity.

### TH uptake assay (MCT8)

The potential inhibition of MCT8-mediated TH uptake was tested as previously described^35^ with slight modifications. Briefly, cells (mdck1-hMCT8) were cultured in DMEM (including 10% fetal bovine serum (FBS), penicillin/streptomycin and G418 for selection) and seeded (20,000 cells/well) in microtiter plates (TTP, Switzerland). After 24 h, the medium was changed to FBS-free and the uptake assay was performed after a further 24 h in culture.

Uptake buffer was added to the cells together with the respective test compound/concentration and T3, resulting in a final concentration of 10 µM T3 on the cells. Silychrstin^36^, a well-characterized MCT8 inhibitor, was used as a positive control (10 µM). Plates were incubated at 37 °C for 20 min, after which the supernatant was removed and the cells washed with PBS (containing 0.1% BSA) and ice-cold water. After the addition of 50 µl ammonium persulfate (0.6 M), the plates were sealed and heated in an oven at 90 °C for 1 h. Finally, the plates were removed from the heating device, cooled to room temperature and the digested cells/released iodide was further diluted 4-8 fold to a total volume of 50 µl in a new microtiter plate. The iodide (released from the digested T3 in the cells) was then quantified photometrically as described above. Changes in absorbance (415 nm) after 20 min were used to calculate individual uptake in %. Solvent control defined 100% activity, while data from SC-treated samples were used to define 0% activity.

### Statistical analyses

Differences between several individual treatments and the control were compared by ANOVA and the non-parametric Kruskal–Wallis test followed by Dunnett’s post-hoc test by GraphPad Prism (Version 9.3.1, (471) or Systat Software Inc. 2008 (Version 11.0). Histopathological results were analyzed for statistically significant differences were compared with the negative control and the treatment groups by Fisher's exact test using SigmaPlot for Windows software (Version 11.0, Systat Software Inc. 2008). Differences were considered statistically significant if *(p ≤ 0.05).

## Results

### Thyroid hormones and TSH in serum

**Figure 2:**
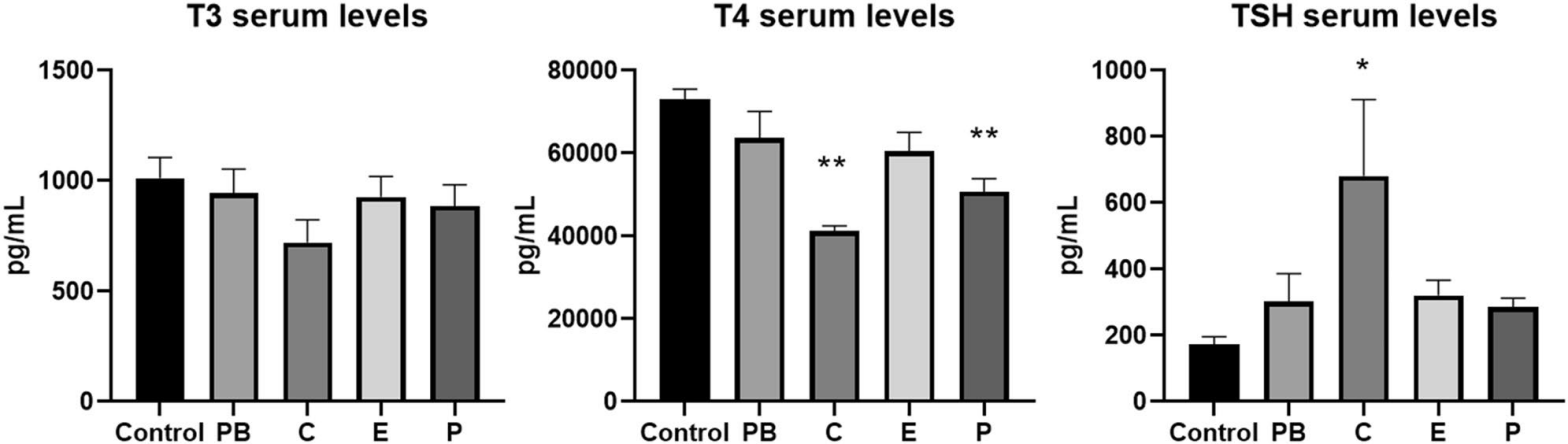
Thyroid hormones and TSH levels in serum of rats treated with selected triazoles and collected after 28 days of treatment (Control: non-treated animals, 6 animals in duplicates measurements; PB: Phenobarbital 500 ppm, 5 animals in duplicates measurements; C: Cyproconazole 1000 ppm, 5 animals in duplicates measurements; Epoxiconazole 900 ppm, 5 animals in duplicates measurements; P: Prochloraz 1000 ppm, 5 animals in duplicates measurements). Values are given as mean ± standard error of mean (SEM). One-Way-ANOVA post hoc (Dunnett’s test) with *(p ≤ 0.05) and ** (p ≤ 0.01).

A statistically significant decrease in serum T4 levels (43.4%) and an increase in TSH levels (293.4%) were observed in rats treated with cyproconazole 1000 ppm when compared to the non-treated animals (Control) (Fig. 2). Prochloraz exposure also significantly decreased T4 levels (30.5%). This effect was less evident at epoxiconazole 900 ppm group. T3 levels were not statistically affected by the treatment, although slightly reduced at cyproconazole group. TSH was statistically increased at cyproconazole application, but not significantly for the other two substances (epoxiconazole and prochloraz). PB treatment related changes in serum T3, T4 and TSH levels were not obvious in this study, but were in line with other similar investigations^37,38^.

### UGT enzyme activity

**Figure 3:**
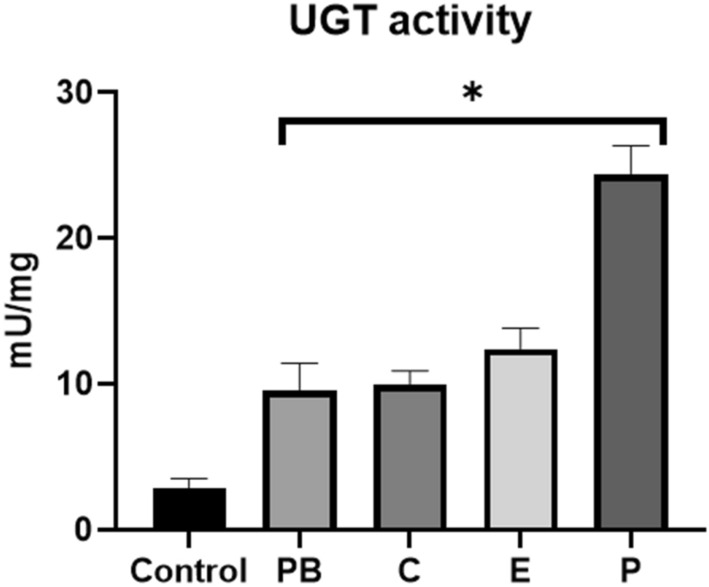
Specific UGT activity across treatment groups (Control: non-treated animals, 6 animals in duplicates measurements; PB: Phenobarbital, 5 animals in duplicates measurements; C: Cyproconazole 1000 ppm, 5 animals in duplicates measurements; E: Epoxiconazole 900 ppm, 5 animals in duplicates measurements; P: Prochloraz 1000 ppm, 5 animals in duplicates measurements) after 28-days of treatment. Data are expressed as mean ± standard error of mean (SEM). One-Way-ANOVA post hoc (Dunnett’s test) with *(p ≤ 0.05).

A subgroup of hepatic UGT-enzymes, known for their ability to conjugate T3 or T4 were induced by cyproconazole, epoxiconazole and prochloraz exposure (Fig. 3). The UGT-enzymes used were a multi-isozyme substrate that is glucuronidated by virtually all mammalian UGT1A and UGT2B enzymes.

### Thyroid histopathology and morphometry analysis

In general, follicular hypertrophy and hyperplasia are the most frequently noted alterations in thyroid tissue, occurring as a response to exposure to goitrogenic substances or secondary to a toxic compound inciting a shift in the HPT axis^5^. Hypertrophy refers to an increase in the size of cells, leading to the enlargement of the organ. The total number of cells in the organ or tissue does not change in hypertrophy; rather, individual cells become larger^39^. Hyperplasia is the process by which there is an increase in the number of cells in an organ or tissue. These extra cells are produced by a higher rate of cell division. This can lead to the enlargement of the organ or tissue, but unlike hypertrophy, this is due to an increased number of cells, not an increased cell size^40^. Hyperplasia in follicular cells can manifest either as diffused, uniformly spread throughout the tissue, or multifocally, concentrated in distinct identifiable areas^41^. It is a reaction of the follicular epithelium to hormonal fluctuations causing disruptions in the feedback mechanism of thyrotropin-releasing hormone and thyroid-stimulating hormone, or due to iodine deficiency^42,43^. The number of follicular cells per unit area can increase in the thyroid, due to the epithelium's papillary infoldings or the stratification of cells lining the follicle^27^. Affected follicles could appear small with scant colloid or larger and irregular with papillary projections of the hyperplastic epithelium extending into the follicular lumen^44^. In the case of follicular cell hypertrophy, the epithelium of the thyroid follicle may present a range from cuboidal to columnar, with central follicles appearing densely packed and smaller than usual^39^. Bilateral hypertrophy typically occurs in response to elevated pituitary secretion of TSH. Hypertrophic follicular epithelium may display pale eosinophilic cytoplasm and small, clear vacuoles. In chronic follicular epithelial hypertrophy, most follicles are smaller than normal, exhibiting secretory depletion or abnormal secretion and exfoliated epithelium^45^.

**Figure 4:**
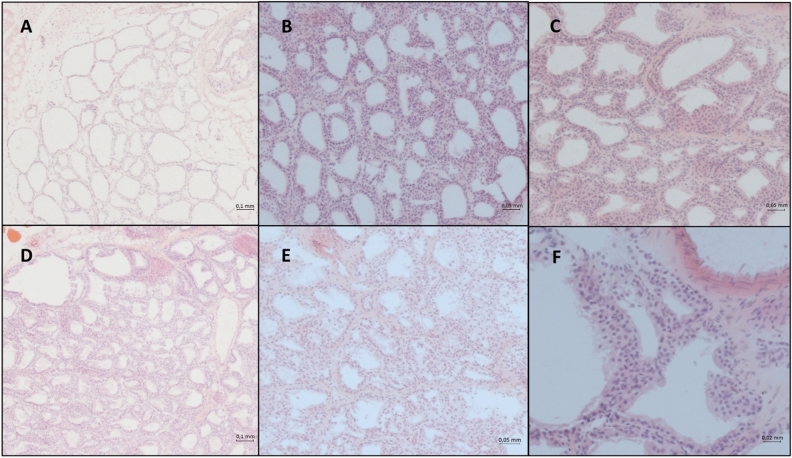
Thyroid follicles at different treatment groups. Optical microscopy (50 × , 100 × and 200 × magnifications), H&E staining. (**A**) Control negative (5×); (**B**) Cyproconazole 1000 ppm (10×); (**C**) Epoxiconazole 1000 ppm (10×); (**D**) Phenobarbital positive control (5×); (**E**) Prochloraz 900 ppm (10×); (**F**) Epoxiconazole 1000 ppm (20×).

Overall, there was follicular dilation observed in the thyroid gland in most of the animals of this study, including the negative control. Thyroid follicular hyperplasia and hypertrophy were evident in rat in treated groups (Fig. 4A–F). The incidence of thyroid follicular hypertrophy, follicular hyperplasia, and follicular dilation was high (80–100%) and observed in all treatment groups (Table 2). The triazoles groups had similar incidence of thyroid follicular changes as PB treated group, a well known thyroid and hepatotoxic substance. The severity of thyroid follicular cell hypertrophy and folicle epithelium hyperplasia varied from focal to disseminated with highest severity grades in the positive control group, followed by the treated groups and lowest severity grades (focal to bifocal) in the untreated control group (Table 3).

**Table 2:** Summary of histological results of rat thyroid tissue at study termination expressed as the number of rats with pathological findings per total number of rats examined (incidence and %) per treatment group.

Incidence	Number of animals observed	Follicular hypertrophy	Follicular hyperplasia	Follicular dilation	No visible lesions
Control negative	6	0	0	4 (67%)	2 (33%)
Phenobarbital 500 ppm	5	5 (100%)*	5 (100%)*	5 (100%)	0
Cyproconazole 1000 ppm	5	5 (100%)*	4 (80%)*	5 (100%)	0
Epoxiconazole 900 ppm	5	5 (100%)*	5 (100%)*	5 (100%)	0
Prochloraz 1000 ppm	4	4 (100%)*	4 (100%)*	4 (100%)	0

*(p ≤ 0.05), Fisher’s Exact Test.

**Table 3:**
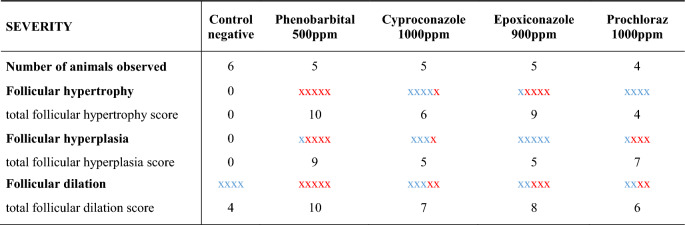
Summary of histopathological results of rat thyroid follicular hypertrophy, hyperplasia, and dilatation at study termination expressed as the number of rats with pathological findings per number of rats examined (incidence notified by an "x") and strength/severity of the findings (effect notified by color code and score) for each substance.

Score system; : slight = 1 point; hyperplasia: one to two follicles affected, hypertrophy: not all follicles or only part of the follicles affected. : moderate = 2 points; hyperplasia: more than one follicle affected (multifocal), hypertrophy: every follicle or almost all follicles affected.

At PB group, follicular cell hypertrophy was observed in all animals. Hypertrophic follicles showed a reduced lumen at the center of the gland (Fig. 4D). Follicular cell hyperplasia occurred as diffuse or disseminated lesions consisting of multiple round-to-oval follicles of various sizes that were lined by multiple layers of cuboidal epithelium; additionally occasional intrafollicular infoldings were observed.

Hypertrophy with occasional hyperplasia formations were observed in all animals exposed to triazoles (Table 2). All animals treated with the azole fungicides showed a follicular epithelium hypertrophy and focal follicular hyperplasia to a certain degree, similar to the effects of PB and clearly showing higher severity than the negative control (Table 3).

**Figure 5:**
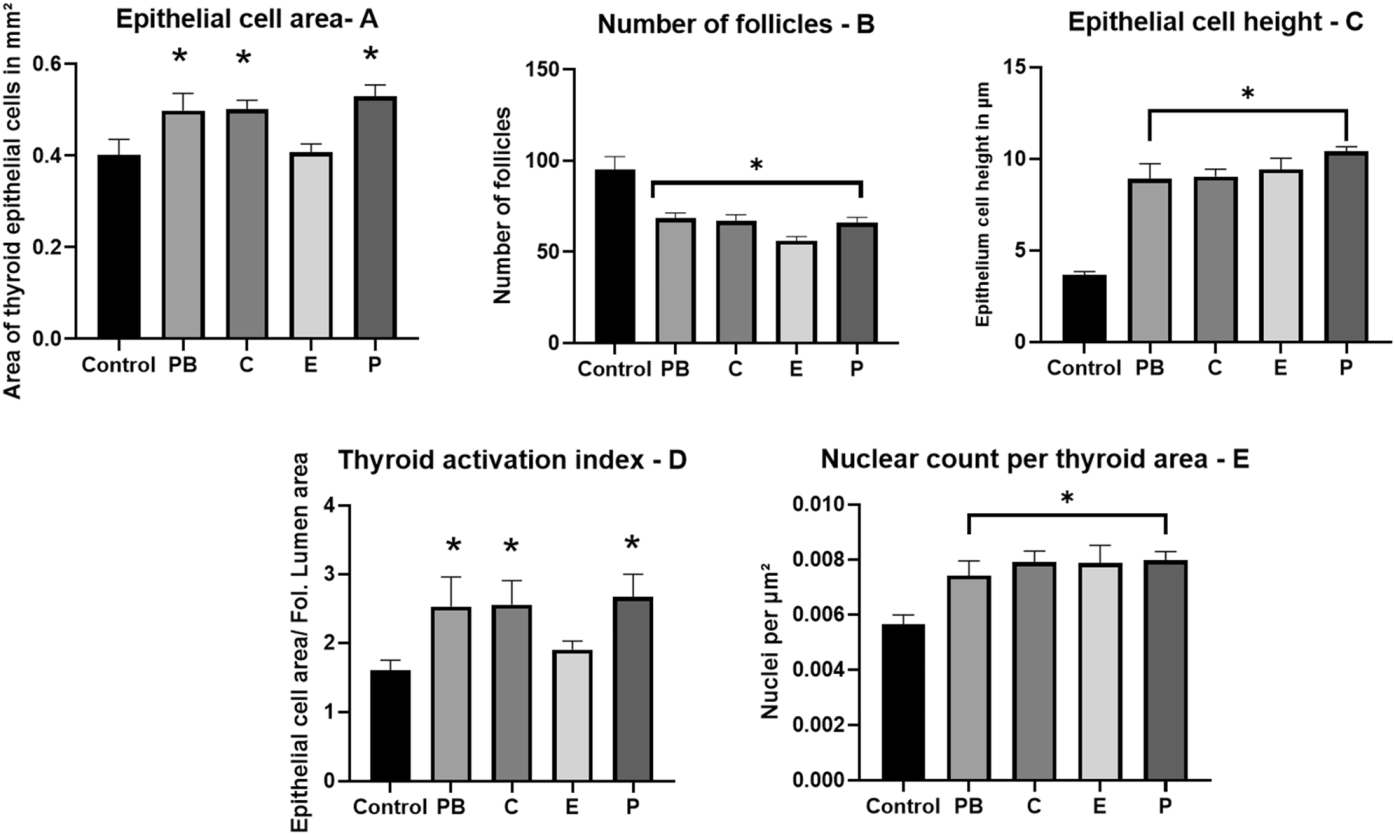
Thyroid morphometric parameters investigated at different treatment groups after 28-days treatment. Control: non-treated animals, 6 animals in duplicates measurements; PB: Phenobarbital, 4 animals in duplicates measurements; C: Cyproconazole 1000 ppm, 5 animals in duplicates measurements; E: Epoxiconazole 900 ppm, 4 animals in duplicates measurements; P: Prochloraz 1000 ppm, 5 animals in duplicates measurements. Values are given as mean ± standard error of mean (SEM). One-Way-ANOVA post hoc (Dunnett’s test) with *(p ≤ 0.05).

The noticeable hypertrophic and hyperplastic changes under the influence of the fungicides and PB could also be confirmed with morphometry analysis in the following relevant thyroid parameters (see also Table 1 for glossary and Fig. 5A–E):

**Epithelial cell area (L2–L3) (**Fig. 5A**):** The average cell area seems to be increased for most of treatment groups compared to the negative control. This could indicate hypertrophic or hyperplastic effects on the cells due to the increased area covered by epithelial cells.

**Number of follicles (**Fig. 5B**):** The average number of follicles decreases under all treatment groups compared to the negative control. This could suggest potential effects on follicle development or maintenance but can be interpreted as an increase in cell size and number—hypertrophic or hyperplastic effects which affects the follicle lumen and negatively changes the number of follicles (decreased).

**Epithelial cell height-L4 Average (**Fig. 5C**):** The epithelial height appears to be significantly increased under all treatment groups, suggesting a potential hypertrophic effect. This data implies a stimulation of the TSH pathway. A stagnation in cell size increase can be explained by a biological limit of the individual cell or due to temporal aspects of substances exposure, i.e. the study period of 28 days might not have been sufficient to capture the full extent of the substances' effects. It's conceivable that with prolonged exposure, the hypertrophic and hyperplastic changes might become more pronounced or even evolve in character, e.g. neoplasia.

**Epithelial cell area by follicle lumen area or “Thyroid activation index” (**Fig. 5D**):** The average ratio increases for most of the fungicides compared to the negative control. This could indicate hyperplasia or increased cell proliferation. It is commonly accepted that this value represents thyroid activity indicating a higher activation of the thyroid^46,47^. This might suggest the existence of complex interactions between the different fungicides and potentially leading to more severe effects.

**Nuclear Count per Area (**Fig. 5E**):** The average nuclear count per area is higher for the treatment groups compared to the negative control. This can be explained with an increase in cell proliferation (hyperplasia). This result indicates that even in 28 days of treatment first indicators for hyperplasia can be observed. Also of note, the increase in cell number appears to be similar in the treatment groups. These results indicate that the underlying pathway for hyperplasia in the thyroid differs from the one for hypertrophy and both effects must be analyzed not only individually, but also in comparison to each other.

Treated animals showed a large delta in the response leading to a higher SEM which can be a result of a high individual response do the administered fungicides.

### Local activities of Dio1 and Dehal1 in Cyproconazole-treated rats

**Figure 6:**
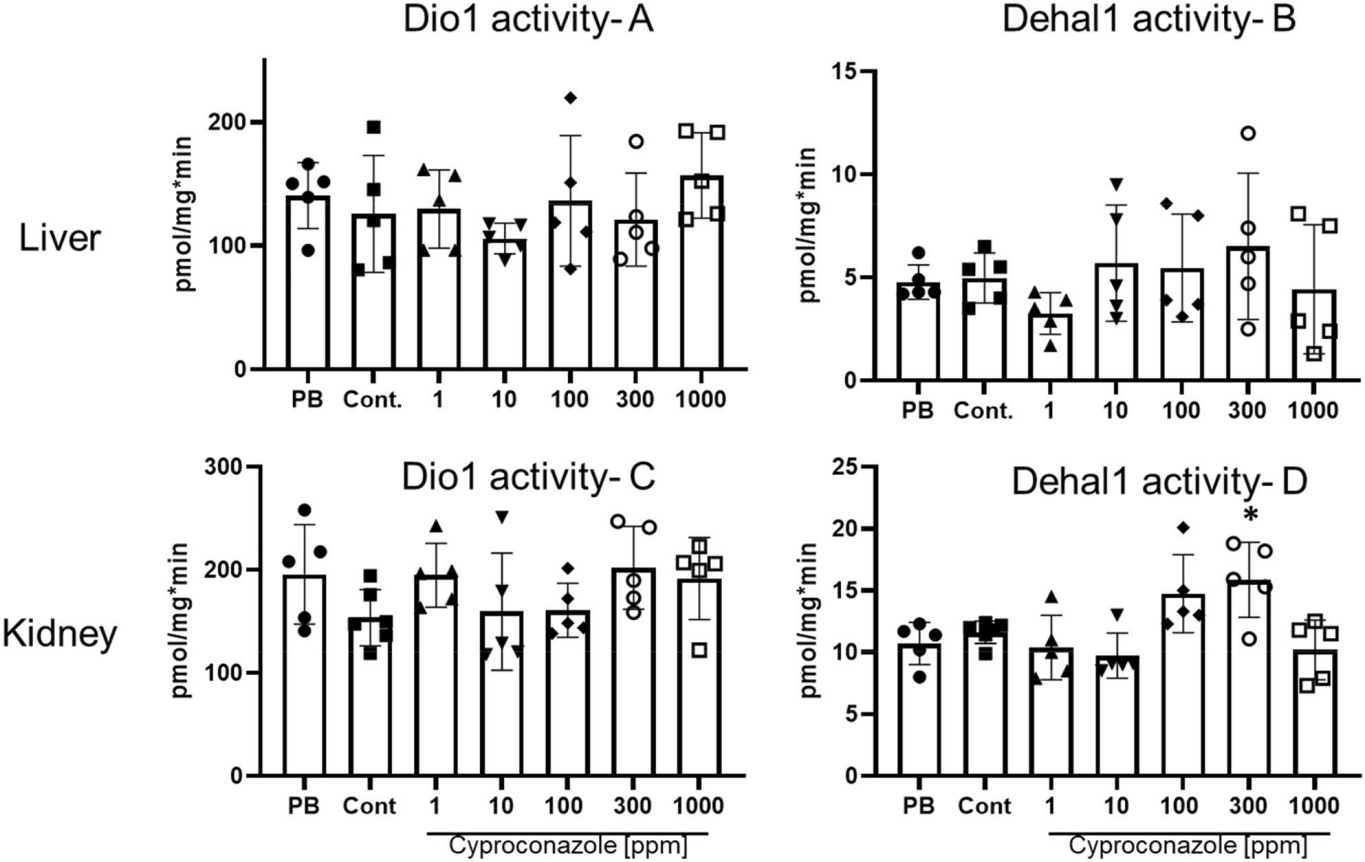
Dio1 and Dehal1 activities in liver and kidney of rats treated with cyproconazole at different doses or PB (Phenobarbital 500 ppm) (mean ± SD, n = 5/group/duplicates); (**A**) hepatic Dio1 activity; (**B**) hepatic Dehal1 activity; (**C**) renal Dio1 activity; (**D**) renal Dehal1 activity. All measurements used the Sandell-Kolthoff-reaction as readout. One-Way-ANOVA post hoc (Dunnett’s test) with *(p ≤ 0.05).

Dio1 and Dehal1 activities were measured from hepatic and renal ex vivo tissue, derived from the 28-days feeding study (Fig. 6A–D). No significant changes were seen for both activities in liver and kidney, except for Dehal1 activity in the kidney of animals treated with cyproconazole 300 ppm only (p = 0.027) (Fig. 6D).

### Summary for in vitro assays

To identify and evaluate a potential interaction of the tested azole fungicides with specific, HPT-related molecular mechanisms, a selection of available in vitro/in chemico assays was used. Tests were conducted to identify interaction of cyproconazole, epoxiconazole and prochloraz with the function of DIO1, 2 and 3, representing relevant local modulators of TH action. Potential interaction with MCT8 as TH transporter was tested, also with TPO activity and function of the NIS transporter, representing two key mechanisms of TH biosynthesis. Furthermore, interaction with DEHAL1 activity was tested, being responsible for efficient iodine retention/recycling in the human (and rodent) organism.

**Figure 7:**
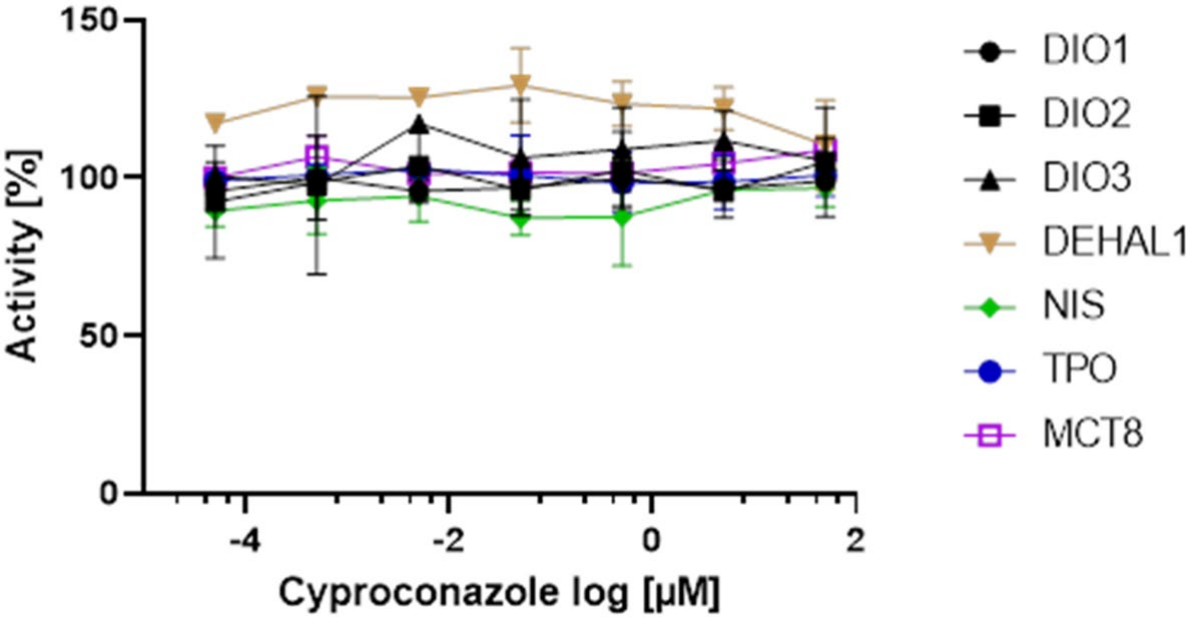
Testing of cyproconazole in selected in vitro assays reflecting potential MIEs. No obvious effects were seen for DIO1-3, DEHAL1, NIS, TPO and MCT8 function up to the maximal tested concentration of 50 µM (mean + /-SD, N = 2, n = 2). Data on epoxiconazole and prochloraz, including data on positive controls, are found in the supplement (SFig. 1).

A summary of the in vitro results for the triazoles is presented at Fig. 7 (cyproconazole) and in the supplement (SFig. 1A,B). Within two independent runs, no consistent, dose-dependent inhibition above 30% was seen for the tested enzymes and transporter activities up to a concentration of 50 µM of cyproconazole. Similarly, for none of the in vitro/in chemico assays, a strong and dose-dependent inhibition was seen when the systems were exposed to either epoxiconazole or prochloraz in concentrations up to 50 µM.

## Discussion

In the present study we combined results from a 28-day feeding study in rats on three different pesticides with a selection of assays in vitro, in chemico and ex vivo to investigate the mode of action leading to the effects on thyroid hormone levels and histopathological changes.

Our findings show an induction of hepatic UGT activities across all treatment groups, which lead to the observed histopathological effects. On the other hand, no dysregulation of the THS-specific enzymatic biomarkers (Dio1 and Dehal1) in liver and kidney of these rats became evident. As these local enzymes, in particular Dio1, are potential indicators of a strongly deranged THS^48^, the absence of a significant regulation might indicate a limited local transmission of the seen hormonal changes to these tissues. Of cause, given group size and strong biological scattering of these activities might limit this statement to strong effects, comparable with reference conditions of hypo- or hyperthyroidism^49^. Furthermore, no indication for specific and highly potent interaction was seen on the level of tested MIEs in vitro. This strongly supports the conclusion that UGT liver enzyme induction would be the major underlying MoA of T4 decline provoked by cyproconazole (and respective TSH induction). This points to the effect on thyroid hormone levels being secondary to hepatic enzyme induction.

Regarding the TH levels, an overall decrease in T4 and an increase in TSH was observed across all treated groups, but significance was not always observed. A statistically significant decrease in serum T4 and increase in TSH levels were observed in test group treated with cyproconazole. Significant decrease of T4 was also observed in prochloraz. Although prochloraz is mainly known to have antiandrogenic/antiesterogenic properties, it has also affected the thyroid in rats by induction of serum T4 decrease and TSH increase in the Hershberger assay at 100 and 200 mg/kg^50^. Concomitantly, it retarded the TH-concentration dependent growth of the rat pituitary GH3 cell lines^51^. Also in amphibians, prochloraz induced circulating thyroid hormone level changes followed by thyroid follicle hypertrophy at the Larval Amphibian Growth and Development Assay (LAGDA) up to 180 μg/L treatment and also on the tadpoles of the Common frog *Rana temporaria* where follicles with rather thick epithelia, follicular cell hyperplasia and colloid depletion were reported up to 251.5 μg/l prochloraz exposure^52,53^.

The observed histopathological changes in the thyroid tissue were follicular hypertrophy, hyperplasia and dilation in all treatment groups. This pathological phenotype could be explained by the activation of thyroid gland due to elevated TSH levels. These observations are in line with findings from the Cumulative Assessment Group (CAG) establishment of pesticides for their thyroid effects report from EFSA^54^ for cyproconazole and in the Danish Environmental Agency study^55^. Studies on similar substances (including azole groups) in the CAG report on thyroid from EFSA in 2019 and other literature also confirm the concern^8,54,56^.

Despite all the three substances showing some similarities with respect to thyroid histopathology there were also pronounced differences in terms of changes in hormone levels. This was also the case in our reference item phenobarbital, as the TH levels were changed slightly (compared to control item) but not significantly, yet the histopathology result was the most pronounced. A potential explanation is the different potency in hepatic enzyme induction by phenobarbital, which has also been described in previous studies. While cyproconazole is the most potent substance, epoxiconazole and prochloraz are less potent with respect to rodent hepatotoxicity^20,21^.

Additionally, according to the OECD 407 protocol (2008) the international evaluation of the endocrine related endpoints (e.g. determination of thyroid hormones (T3, T4) and TSH) could not be demonstrated and is an optional measurement in five males and five females animals per dose level after 28-days exposure. Therefore, as confirmed in our study “definitive identification of thyroid-active chemicals is more reliable by histopathological analysis rather than hormone levels”^26^. Future research should explore underlying mechanisms, long-term effects, and potential cumulative impacts, considering longer exposure periods. A greater sample size, multiple time points of collection, uniform historical data from with various commercial assay kits are also some of the improvements necessary to understand individual responses to active substances.

Overall, there is no clear guideline or protocol which determines how thyroid tissue can be successfully analysed. The data gathered indicates that the typically observed limited scale of combination effects in conventional toxicology may stem from methodological constraints. Specifically, histopathology yields dichotomous data instead of continuous data. However, this limitation can be addressed using a morphometric approach as it provides quantifiable and objective data on structural alterations in the tissue, which is critical for understanding the toxicological implications of these substances. But nevertheless, it should be always combined with molecular analysis and in vitro test for assessing the exact mechanism. The tabulated data and pictures clearly show an increase of epithelial cell volume and an increase in nuclei per area which indicates the presence of hyperplasia and hypertrophy. Also, there is evidence of epithelium cell height which indicates a stimulation of the TSH pathway. A stagnation in cell size increase can be explained by a biological limit of the individual cell, particularly considering the short treatment duration of 28 days. In summary, signs of hypertrophy and hyperplasia observed via classical histological analysis were confirmed by morphometric analysis standardized at this study conditions.

With regard to the fungicides MoA, potential explanation of the effects observed in the thyroid tissue is the substance-related induction of phase II hepatic enzymes like UGTs^57–60^. The potential molecular initiating events of the respective pathway consist of nuclear receptor activation, such as CAR. Seeger et al.^61^ confirmed that cyproconazole activates CAR in human and rat liver in vitro cell lines. Additionally, at public databases (US EPA ToxCast), there are in vitro tests showing activity for epoxiconazole and prochloraz on the human thyroid hormone receptor alpha (THRA) and beta (THRB) genes in GH3, a rat pituitary gland cell line^62,63^. There are no THR test results for cyproconazole at this platform but cyproconazole and prochloraz were tested positives for activity on the thyroid hormone responsive gene THRSP in HepaRG cells (human liver cell line) and HEK293T, respectively^64^. Prochloraz was also tested and active in a Thyrotropin-releasing hormone receptor (TRHR) antagonist assay in HEK293T^63^. In these studies transcriptomics and metabolomics also confirmed that similar toxicity pathways are affected by these pesticides and other azoles, thus indicating similar MoA.

Also an important discussion in risk assessment of thyroid effects is, whether or not the corresponding effects are relevant to humans^65^. While nuclear receptors mediate drug-induced changes in the expression of drug-clearance pathways, the species differences in nuclear receptors activation make the predictions difficult to assess^8,66^. Generally, substances inducing hepatic conjugation (and therefore inactivation) of thyroid hormones via CAR or PXR are considered to be of questionable relevance to humans as the sensitivity of human and rodent nuclear receptors CAR or PXR responsible for this induction differs substantially for many substances^67,68^. Activation of these receptors by xenobiotics has been linked to hepatotoxic effects as well as promoting hepatocellular adenomas and carcinomas through non-genotoxic pathways in rats^69^. While some studies show that PB is of no human relevance when evaluating hepatocellular tumor induction via non-genotoxic mechanisms^67,70^, others challenge this idea with the recent inclusion of ‘humanized mouse cells’ that express these receptors^69,71^. Therefore the discussed “non-relevance” can be misleading, as this argument can only question the sufficiency of the rodent model but not exclude the potential adverse effects in humans. This aspect is also discussed at the current^16^ where the rat liver mediated MoA cannot be considered as non-human relevant without appropriate evidence. Additionally, “the hazard assessment of a substance should consider the most sensitive population and reductions in T4 levels should act as a trigger for further studies of F1 generation (e.g. as part of most updated OECD test guidelines 421/422, 426,416, 443) (OECD, 2001, 2007, 2012, 2016a,b) depending on the other information available”^16^.

According to the results from in vivo studies performed in rodents with triazoles, activation of these receptors is discussed to be involved in many adverse liver effects, which also include liver hypertrophy to hyperplasia and induction of liver enzymes^66^. In mice bearing the human form of the receptors CAR and PXR (hCAR/hPXR), an activation was observed, although very weak, after 50 or 500 ppm cyproconazole exposure during 28 days^9^. However, a difference was observed when comparing these results in human receptors with the induction in wild-type mice under the same treatment: the staining pattern was more pronounced and expanded from the hepatic perivenous to periportal areas at the wild-type mice^9^. Although these differences were deemed in the past to rule out the relevance of thyroid effects of these triazoles in humans, a more detailed review of enzyme liver induction MoA and metabolism (ADME properties) in different species and humans, as well as the activity of possible metabolites might be advisable as to properly understand potential human relevance^16^.

While in summary this conclusion already raises evidence for the MoA responsible for the observed TH decrease/TSH increase, other molecular initiating events that take a role in TH synthesis (that include e.g. TPO inhibition, NIS inhibition, TSHR binding), serum TH transport, TH receptor activation, cellular TH transport, and peripheral TH metabolism^19^ could be affected in parallel. Therefore, we were aiming to complete the picture by looking at more detailed studies on local endpoints/key events in TH target organs ex vivo and experimental evaluation of a subselection of TH-related MoA by applying cyproconazole (as well as epoxiconazole and prochloraz) to relevant available in vitro and ex vivo test systems.

Our in vitro assay results dedicated to THS specific interaction by cyproconazole, epoxiconazole and prochloraz are supporting the proposed MoA as none of the other targets tested so far were affected (including thyroidal I^–^-uptake (NIS), thyroid peroxidase (TPO), dehalogenase (DEHAL1) and iodothyronine deiodinases (DIO1–DIO3) inhibition). Furthermore, local enzymatic activities of hepatic and renal Dio1 and Dehal1, which are discussed as novel ex vivo endpoints and meaningful indicators in situations of severe hypo- and hyperthyroidism^22^ were unchanged. The significance of the Dehal1 activity in kidney at only one cyproconazole dose is inconsistent and not dose-dependent. This might indicate that the observed changes in T4 and TSH have limited impact concerning the target organs studied until now.

Thus, a mechanistic analysis was important to identify the MoA and/or AOP affected by triazoles, with cyproconazole being the most potent one identified in this study. In this case an endocrine MoA could also be established and can help on further investigations on the endocrine disruption potential of other pesticides.

Regulatory studies usually depend on a subset of in vivo readouts to identify adversities, but to characterize a causal link between an induced adversity and an endocrine MoA, additional ex vivo biochemical assays can help. These assays can be used to close the knowledge gap towards the underlying MoA without the need of additional animals given that the necessary tissues are already available for biochemical investigations as described by Renko et al.^22^. Furthermore, by using “human” in vitro test systems in parallel, additional information on the physiological relevance of in vivo rodent data can be gained to facilitate regulatory decision making. Within the field of THS disruptors, this is of special interest, as the classical in vivo TG protocols just cover hormonal measurements and thyroid histopathology, thereby ignoring aspects of potential dysregulations found at the level of local control of TH action in the target-organs, e.g. by affecting deiodinases or TH –uptake into the target cells.

In conclusion, this study aimed to explore the impacts of fungicide on thyroid function and thyroid hormone metabolism. As part of this effort, mechanistic assays were developed to investigate the MoA of substances known to affect thyroid hormone levels. While the tested substances induced changes in thyroid parameters in our study, the magnitude and nature of these changes varied. Unravelling these interactions could provide valuable insights into the toxicological behaviour of these substances and give more solid foundation for further risk assessment. Still, the application of selected methods for characterization of potential endocrine disruptors inducing changes in TH serum levels by HPT primary and secondary mechanisms generated a much more conclusive dataset, as shown by this study.

### Supplementary Information

Supplementary Figure 1.

## Data Availability

The datasets generated during and/or analysed during the current study are available from the corresponding author on reasonable request.
