# Supplementary material for: In vitro and in vivo investigation of a thyroid hormone system-specific interaction with triazoles
Source: Sci Rep. 2024 Mar 18;14:6503. doi: 10.1038/s41598-024-55019-3 (PMC10948911; doi:10.1038/s41598-024-55019-3)
Supplement: Supplementary file 1 — Supplementary Figure 1. [file 41598_2024_55019_MOESM1_ESM.pptx]

## Slide 1
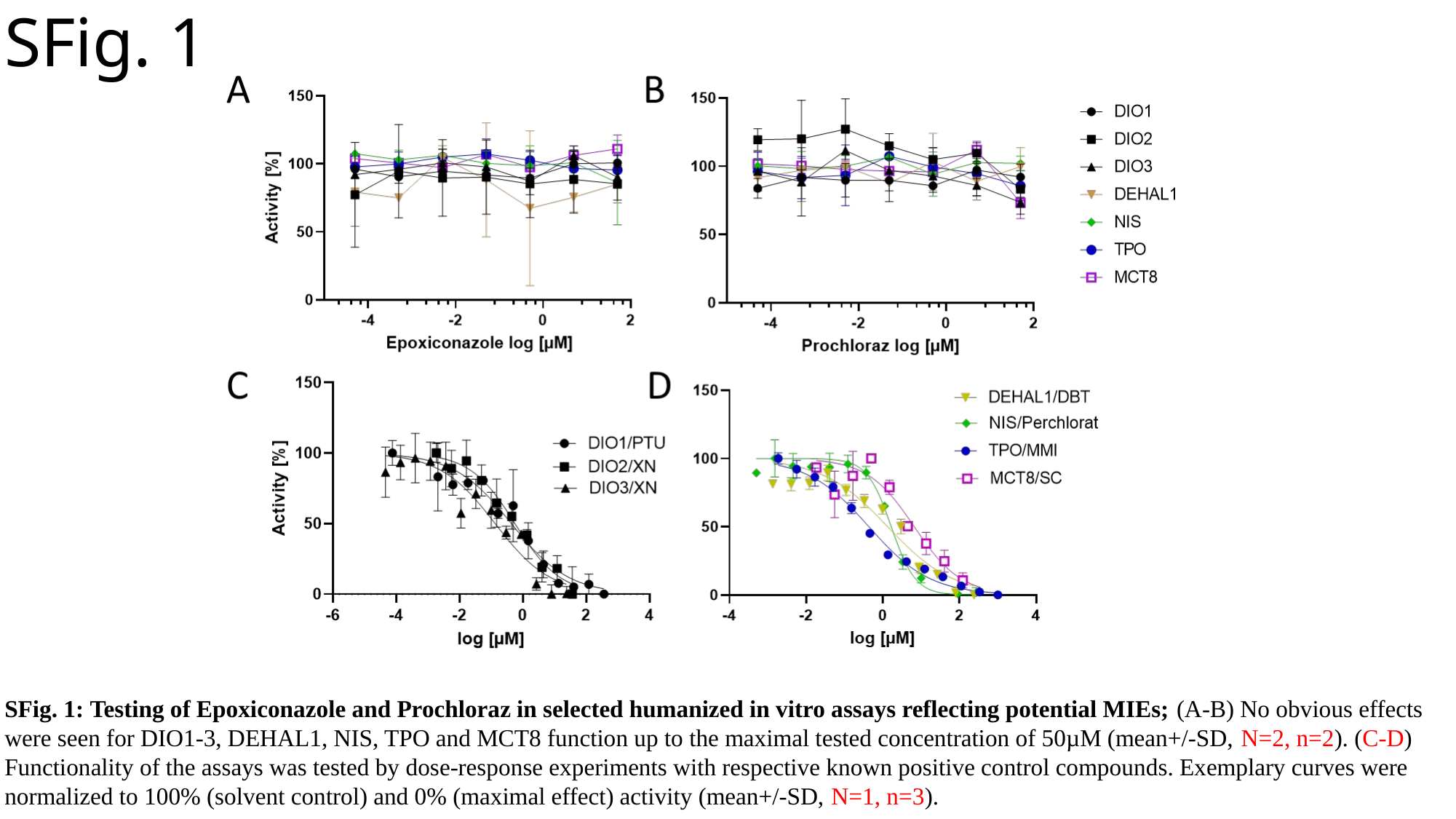

SFig. 1
SFig. 1: Testing of Epoxiconazole and Prochloraz in selected humanized in vitro assays reflecting potential MIEs; (A-B) No obvious effects were seen for DIO1-3, DEHAL1, NIS, TPO and MCT8 function up to the maximal tested concentration of 50µM (mean+/-SD, N=2, n=2). (C-D) Functionality of the assays was tested by dose-response experiments with respective known positive control compounds. Exemplary curves were normalized to 100% (solvent control) and 0% (maximal effect) activity (mean+/-SD, N=1, n=3).
